# Twenty Years’ Development of Teacher Identity Research: A Bibliometric Analysis

**DOI:** 10.3389/fpsyg.2021.783913

**Published:** 2022-02-04

**Authors:** Yunyun Zhang, Ping Wang

**Affiliations:** School of Foreign Languages, Jiangsu University of Science and Technology, Zhenjiang, China

**Keywords:** twenty-year development, teacher identity, bibliometric analysis, identity, construction

## Abstract

This study aims to demonstrate a detailed knowledge map of teacher identity research *via* a 20-year data set from the Web of Science (WoS) database. A bibliometric analysis was employed for analyzing the articles published between 2001 and 2021 to show the status of teacher identity research in the past 20 years, research topics on teacher identity, and future research directions. Using the keyword “teacher identity” and filtering data by selecting articles and early access in teaching and education, 848 articles were retrieved. Through production, content, and citation analysis with the help of a bibliometric tool, this study found that teacher identity remained a popular research theme in the academic field over the past 20 years, and its booming production involved many authors, institutions, and sources, and countries. Furthermore, teachers’ “beliefs,” “emotions,” “professional development,” and “context” impacting the construction and reconstruction of teacher identity were the popular topics in teacher identity research, and fundamental issues, including “identity,” “teacher identity,” “professional identity,” “development,” “teacher development,” “beliefs,” and “intersectionality” of teacher identity keep good topics in future research.

## Introduction

### Teacher Identity Research

The teacher identity has played an essential role in education research and teacher education (Lopes and Pereira, 2012; Jupp and Lensmire, 2016) because the construction of teacher identity is related to the teacher’s professional development, thus impacting their teaching practices and attitudes (Pennington, 2014; Schutz et al., 2018). Moreover, the factors and the context influencing the construction of teacher identity enlighten the teacher education program and school managers to enhance program quality and management. Just as Beijaard et al. (2004) argued that teacher education programs could be improved by gaining a complete understanding of identity generally and teacher identity in particular. Furthermore, we could better design our teacher education programs through learning about teacher identity from the literature.

There is no doubt that academic publications on the development of teacher identity have been booming over the past 20 years, and they contribute to the development of various aspects of teacher identity research. However, it also becomes increasingly unfeasible to keep pace with everything being published simultaneously. In terms of teacher identity research, there are increasingly more studies on the topics related to it, and several reviews related to some specific aspects of teacher identity have been done. However, there has been a dearth of research on a general and an overall picture of teacher identity research in the past 20 years. A bibliometric analysis adopted in this study will give insights into current research status, research topics, and future research directions in teacher identity. We believe that this study is a valuable tool for practitioners and scholars in the research field of teacher identity. Early researchers interested in teacher identity can provide them with the knowledge needed to begin their research. For experienced teacher identity researchers, this study also serves to better understand the field’s development and promote networking and collaboration between institutions and authors.

### Bibliometrics Analysis Tool

Under the background of big data, there is a vast amount of literature in various research fields. However, many studies are scattered and hard to be arranged in an organized and transparent way. Consequently, quickly and accurately finding out the critical literature closely related to the research topic has always been perplexing (Aria and Cuccurullo, 2017). The study of teacher identity is taken as an example. There is too much relevant literature on teacher identity, covering multiple directions. It makes it more difficult for researchers to screen the literature they need the most.

A bibliometric analysis characterized by a quantitative analysis of the articles published in a specific field (Baker et al., 2020) is one of the literature reviewing approaches to accumulate knowledge and organize previous findings. It is a particularly prevalent method when investigating various aspects of science and how institutions and universities are ranked worldwide (Ellegaard and Wallin, 2015). A bibliometric analysis is based on the Bibliometrix package in R, an open-source ecosystem encompassing statistical algorithms, mathematical functionality, and visualization capabilities (Derviş, 2019). At present, the bibliometric analysis is applied to a vast range of research fields, including tsunami research (Chiu and Ho, 2007), in the management and organization (Zupic and Èater, 2015), in tourism (Koseoglu et al., 2016), TB research (Nafade et al., 2018), Economics in Latin America (Bonilla et al., 2015), and the COVID-19 pandemic (Gautam et al., 2020). The bibliometric method in this study has significantly powerful functions and plays an increasingly important role in research thanks to its scalable and reliable statistics. Compared with other approaches, it may introduce a systematic, transparent, and reproducible review process based on the statistical measurement of science, scientists, or scientific activity (Pritchard, 1969; Diodato, 1994). As a particularly suitable method for science mapping, it adapts to when the emphasis on empirical contributions produces voluminous, fragmented, and controversial research streams (Aria and Cuccurullo, 2017). The increasing number of publications applying bibliometric analysis in all fields indicates that it fulfills the need of policymakers, researchers who demand valuable and exact research based on massive literature. In addition to the benefits of researchers, the overall result produced by the bibliometric tool also benefits policymakers by helping them access the performance of scholars and institutions.

It is evident that some review studies on the topic of teacher identity have been done, such as teacher identity in the university context (van Lankveld et al., 2016), non-native English teacher identity research (Yuan and Mak, 2018), teacher educators’ identity (Izadinia, 2014), issues in the teacher identity literature, and implications for teacher education (Beauchamp and Thomas, 2009). This study, however, contributes to teacher identity research by providing a knowledge map of this field. By employing a bibliometric analysis, we hope that this knowledge map will serve as a valuable tool for early researchers to find out knowledge and research findings to begin their research as soon as possible. We also hope that this study serves as an effective way for experienced researchers to understand the development of teacher identity research in the past 20 years, find research gaps that they can fill in the future, and help them find out potential cooperators. Furthermore, we expect that this study will serve as a reliable measure for some rating agencies to evaluate the performance of authors, institutions’ sources, and countries in teacher identity research effectively and quickly.

## Methodology

### Research Questions

The present study aims to identify and provide evidence for the state and impact of teacher identity research in the past 20 years. This study analyzed the research topic, publication pattern, research area, authors, highly cited articles, journals, and institutions that contributed most to teacher identity research in the past 20 years. The following questions were designed to plan the review of teacher identity articles in the Web of Science (WoS) database: (1) What was the research status of teacher identity research in the past 20 years? (2) What were the research topics on teacher identity research in the past 20 years? (3) What are the future research directions in teacher identity research? This study offered productivity, citation, and content analysis to answer these questions, such as the number of documents published and citations, co-occurrence of keywords, most relevant sources, authors and institutions, collaboration network, word cloud, and trend topics.

### Data Sources

In this study, all data were retrieved from the WoS core collection database [including the citation Index Social Sciences Citations Index (SSCI) and Arts and Humanities Citation Index]. WoS is widely used for academic and bibliometric studies as it gives consistent journal coverage of scholarly published articles (Li et al., 2018; Birkle et al., 2020). The data were retrieved on July 16, 2021, and the research term was “teacher identity,” with a published timespan from 2001 to 2021. The literature types were mainly restricted to studies and early access. The literature data obtained included the complete records (the author’s title, source year, abstract, keyword, DOI number, citation frequency, etc.) and the references cited in the article.

### Data Collection and Data Analysis

This research selected the WoS that contained the data from 2001 to 2021, filtered the core document set according to the title of the study, and exported the 848 data from the selected database. Data collection was divided into three substages. The first was data retrieval. This research chose the WoS as a source of bibliographic information. We selected the articles and early access stored in the SSCI and arts & humanities citation index (AHCI) to analyze research questions. The second substage was data cleaning. We checked articles to avoid data repetition. In the third substage, files were downloaded and compressed. We downloaded 500 references the first time and 348 references the second time. Then, the two article files were compressed for employing bibliometric tools.

Nowadays, different tools are available to perform bibliometric studies, including CiteSpace, HistCite, VOSviewer, and CitNetExplorer (Moral-Muñoz et al., 2020). This study adopted a biblioshiny program to acquire a general picture of teacher identity research in the past 20 years. Biblioshiny was developed by Massimo Aria and Corrado Cuccurullo from the University of Naples and the University of Campania’s Luigi Vanvitelli (Hao, 2018). It is powered by Bibliometrix and its web-based graphical interface based on WoS, Scopus, and Dimensions data (Aria and Cuccurullo, 2017). In an up-to-date review on software tools for conducting a bibliometric analysis in science, Moral-Muñoz et al. (2020) have already given an introduction on biblioshiny. According to them, the interface of biblioshiny is intuitive and well organized, and the main menu is divided according to the Science Mapping Analysis (SMA) workflow (Moral-Muñoz et al., 2020). The menu in biblioshiny will conduct performance analysis from source, author, and document dimensions, and conceptual, intellectual, and social structures of knowledge also will be done through this menu. The analysis options are mainly subdivided into eight categories: (1) data set, (2) sources, (3) authors, (4) documents, (5) clustering, (6) conceptual structures, (7) intellectual structure, and (8) social structure. Several kinds of file formats can be exported, maps can be shipped to Pajek and Html, and tables can be copied to the clipboard or saved as excel, pdf, or printed.

The retrieved data in this study were analyzed for bibliometric indicators using the Rstudio software with bibliometric R-package v.4.0.4. First, the bibliometric analysis was activated in the R environment using the R language “install. packages (“bibliometrics,” dependencies = TRUE).” The command code opened a biblioshiny web interface on the google chrome browser. Raw data in WoS were imported into the biblioshiny and analyzed. Then, the author described and interpreted the findings indicated by statistics and pictures, which is a crucial part of this research. This study applied relevant authors, institutions, countries, articles, top highly cited publications, keyword co-occurrence, word cloud, thematic map, trend topics, and conceptual structure to answer three different research questions.

## Results and Discussion

### The Status of Teacher Identity Research in the Past 20 Years

For the first question: what was the research status of teacher identity in the past 20 years? In this study, a productivity analysis was performed. Preliminary information about data, most relevant authors, institutions and documents, countries dominating the teacher identity study, and the co-citation of authors and sources were presented to show the status of teacher identity research in the past 20 years.

#### Main Information About Data

Table 1 presents general and comprehensive teacher identity research from 2001 to 2021. The sources of the teacher identity study involved 238, including journals and books. There were 848 documents in total. The average years from publication, average citation per document, and average citations per year per document were 6.2, 19.48, and 2.197, respectively. Moreover, the reference contained in the articles reached 27,422, which shows the popularity of the study of teacher identity in the past 20 years. The 751 articles accounted for the most significant published document types. In terms of document contents, the keyword plus and the author’s keywords were 805 and 1,773, respectively. It indicates a wide range of research content in teacher identity research. We also know that 1,447 scholars from 2001 to 2021 published articles and contributed to teacher identity. There were 317 authors of single-author documents and 1,130 authors of multi-author articles. The documents per author, authors per document, coauthors per document, and collaboration index were 0.586, 1.71, 2.04, and 2.34, respectively. It suggests that more scholars paid attention to teacher identity research, and cooperation between authors was the most effective means for teacher identity research in the past 20 years.

**TABLE 1:** Main information about data.

Description	Results
*Main information about data*	
Timespan	2001:2021
Sources (Journals, Books, etc.)	238
Documents	848
Average years from publication	6.2
Average citations per document	19.48
Average citations per year per doc	2.197
References	27,422
*Document types*	
Article	751
article; early access	54
article; proceedings paper	8
book review	34
book review; early access	1
*Document contents*	
Keywords plus (ID)	805
Author’s keywords (DE)	1,773
*Authors*	
Authors	1,447
Author appearances	1,734
Authors of single-authored documents	317
Authors of multi-authored documents	1,130
*Authors collaboration*	
Single-authored documents	366
Documents per author	0.586
Authors per document	1.71
Coauthors per documents	2.04
Collaboration index	2.34

**FIGURE 1:**
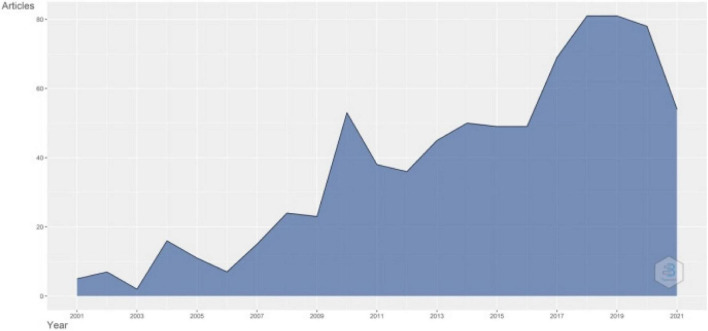
Annual scientific production.

**FIGURE 2:**
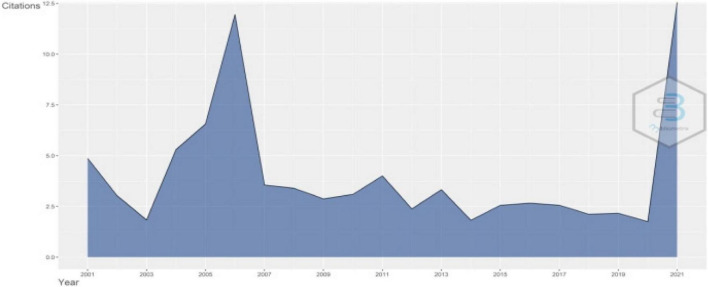
Average citation per year.

The annual production about teacher identity is shown in Figure 1. This study concludes that scholars generally paid increasing attention and output during this period achieved a dramatic growth from less than 10 in 2001 to more than 80 in 2018. The booming research about teacher identity during 2018 indicates a research climax in teacher identity research. Additionally, in recent years, the productivity of teacher identity remained high and stable. We can conclude that teacher identity remained a hot research topic and gained momentum in recent years. From Figure 2, we get that the article citation reached a peak in 2006. It indicates that the study of teacher identity was actually in the preliminary stage, and there were relatively fewer publications. It coincides with Figure 1, which shows fewer annual scientific production in 2006. The stable average citation per year after 2014 in Figure 2 shows that teacher identity studies remained stable recently. Therefore, we can conclude that teacher identity research was in a phase of steady growth.

#### Most Relevant Sources

Table 2 presents the top 20 relevant sources, which considered teacher identity and topics related to it as important research content and theme. It suggests that the journal *Teaching and Teacher Education* was the essential research front of teacher identity as it published 106 relevant articles and has 38 h-index, 78 g-index, 2.1 m-index, and 6,175 total citations in the past 20 years. Followed by 34 in *Teachers and Teaching* and 30 in *European Journal of Teacher Education*, it is proved that the journal *Teaching and Teacher Education* was far away ahead in all aspects of teacher identity research and the journal *Teachers and Teaching* and the journal *European Journal of Teacher Education* were also the two essential research fronts after *Teaching and Teacher Education* in teacher identity research in the past 20 years. Teacher identity was undoubtedly a hot topic in this study indicated by the fact that increasing more journals devoted themselves to teacher identity research. From Table 3 of Source Growth, we can see that many journals paid much more attention to teacher identity research. Among them, the publication of *Teaching and Teacher Education* increased more quickly than the publication in other journals. It coincides with that this journal served as a leading role in teacher identity research in the past 20 years.

**TABLE 2:** Most relevant sources.

Sources	Articles	H-index	G-index	M-index	Total Citation
Teaching and Teacher Education	106	38	78	2.1	6,175
Teachers and Teaching	34	14	27	1.2	735
European Journal of Teacher Education	30	11	22		598
Journal of Language Identity and Education	28	5	6		77
Journal of Education for Teaching	23	7	14	0.5	217
Asia-Pacific Journal of Teacher Education	20	7	15	0.5	253
TESOL Quarterly	19	12	17	0.75	674
Research Papers in Education	14	7	11		134
System	13	4	8	0.3	104
Asia Pacific Journal of Education	11	4	8		67
Gender and Education	11	5	9	0.25	138
Journal of Teacher Education	10	7	10		174
South African Journal of Education	10	4	6	0.2	49
Teachers College Record	10	5	9	0.2	159
British Educational Research Journal	9	7	8	0.3	636
Modern Language Journal	9	6	6	0.5	201
International Journal of Bilingual	8	4	6		45
Science Education	8	6	7	0.3	451
Educational Review	7	4	5	0.2	64

**TABLE 3:** Source growth.

Year	Teaching and Teacher Education	Teachers and Teaching	European Journal of Teacher Education	Journal of Language Identity and Education	Journal of Education for Teaching	Asia-Pacific Journal of Teacher Education	TESOL Quarterly	Research Papers in Education	System	Gender and Education
2001	0	0	0	0	0	0	0	0	0	0
2002	0	0	0	0	0	0	0	0	0	1
2003	0	0	0	0	0	0	0	0	0	2
2004	3	0	0	0	0	0	0	0	0	2
2005	8	0	0	0	0	0	0	0	0	2
2006	11	0	0	0	0	0	1	0	0	2
2007	11	0	0	0	0	0	2	0	0	2
2008	13	0	1	0	0	0	3	0	0	2
2009	19	0	2	0	0	3	3	0	0	2
2010	34	0	5	0	1	3	4	1	0	3
2011	40	2	6	0	3	4	4	1	1	4
2012	43	4	9	0	3	4	6	1	1	4
2013	48	7	12	1	4	6	6	2	1	5
2014	50	10	13	3	4	7	6	2	1	7
2015	56	13	15	4	5	11	7	4	2	9
2016	62	17	15	8	5	13	14	6	3	9
2017	73	22	15	11	10	16	15	7	4	9
2018	81	23	18	12	13	18	16	9	8	9
2019	94	30	19	16	15	18	16	10	11	9
2020	100	31	19	24	20	18	18	11	12	11
2021	106	31	22	25	23	19	19	12	13	11

#### Most Relevant Authors

Table 4 presents the top 20 critical persons in the research field of teacher identity by presenting their document numbers, index, local citation, and total citation. John Trent was the most significant researcher with 17 articles and 11 h-index, 16 g-index, and 0.8 m-index. John Trent was critical in exploring many topics related to teachers’ professional identity in the past 20 years. For instance, he studied teacher education as identity construction from action research (Trent, 2010), and the practice, language, and identity in a teaching practicum from learner to teacher (Trent, 2013), the native-speaking English teachers, and educational discourse in Hong Kong (Trent, 2012) and other relevant topics about teachers’ professional development. Rui Yuan followed John Trent with 11 articles and 5 h-index, 10 g-index, and 0.7 m-index. Rui Yuan played an essential role in understanding pre-service teachers’ professional development, motivation, and emotion (Yuan and Lee, 2014; Yuan and Mak, 2018) and student–teachers (Yuan and Burns, 2016; Yuan and Lee, 2016). Following Rui Yuan, Sonja Lutovac published 8 articles with 4 h-index, 6 g-index, and 0.3 m-index. Reviewing his study, we can conclude that he paid much more attention to teacher identities in mathematical education, such as pre-service teachers’ future-oriented mathematical identity work, pre-service elementary school, and mathematics teachers’ narrated possible selves about how failure shapes teacher identities. We can conclude that these three authors were the most prolific and fundamental in teacher identity research. Beijaard and Meijer (2017) were also the key authors in terms of local citation and total citation. It was proved that they were essential scholars and researchers in studying teacher identity, and their theories and views might provide a theoretical and practical framework for further research. It was around these authors that research circles of teacher identity had been shaped. Most of the other authors had relatively average four to five articles and a similar index, which shows that they were also indispensable persons promoting the further development of teacher identity research.

**TABLE 4:** Most relevant authors.

Authors	Articles	H-index	G-index	M-index	Local citation	Total citation
Trent J	17	11	16	0.8	29	268
Yuan R	11	5	10	0.7	23	117
Lutovac S	8	4	6	0.3	23	88
Beijaard D	6	5	5	0.2	309	1,296
Den Brok P	6	5	5	0.5	47	191
Kaasila R	6	5	5	0.4	24	103
Zhu G	6	3	4		3	35
Han I	5	2	3	0.3	11	20
Lofstrom E	5	4	5	0.3	26	107
Meijer PC	5	3	3	0.16	349	1,385
Menard-Warwick J	5	3	3	0.2	0	85
Van Der Want AC	5	3	4	0.4	12	26
Zhu J	5	2	4		0	16
Archer L	4	4	4	0.2	0	119
Avraamidou L	4	4	4	0.5	27	101
Ballantyne J	4	3	4	0.3	9	52
Juzwik MM	4	4	4	0.3	12	98
Lee I	4	4	4	0.4	25	121
Lee JCK	4	2	4	0.2	1	27
Lopes A	4	3	4	0.2	5	55

#### Most Relevant Affiliations

Table 5 presents the top most relevant affiliations according to the number of articles about teacher identity. The Hong Kong Institution of Education, Education University of Hong Kong, and the University of JYVASKYLA were the three most relevant affiliations by producing 38, 30, and 29 articles about teacher identity in the past 20 years. Many famous scholars in these institutions devoted themselves to explore related topics and contribute to teacher identity research development. The development of teacher identity research in the past 20 years resulted from the joint efforts of many institutions that focused on issues in teacher identity.

**TABLE 5:** Most relevant affiliations.

Affiliations	Articles
Hong Kong Inst Educ	38
Educ Univ Hong Kong	30
Univ Jyvaskyla	29
Monash Univ	22
Univ Sydney	22
Eindhoven Univ Technol	20
Michigan State Univ	18
Univ Auckland	18
Univ S Florida	18
Columbia Univ	17
Univ Autonoma Barcelona	17
Univ Helsinki	17
Univ Hong Kong	17
Univ Oulu	17
Deakin Univ	16
Univ Missouri	16
Texas AandM Univ	15
Univ Porto	14
Univ Texas Austin	14
Chinese Univ Hong Kong	13

#### Most Relevant Countries

Table 6 presents the top 20 relevant countries’ publications, total citation, and average article citation. According to Table 6, we can get that the United States contributed most to teacher identity research with 639 publications, 4,096 total citations, and 21.52 average article citations. They were followed by China and United Kingdom with 238 and 236 journals. It suggests that the United States, China, and the United Kingdom were the top three countries exploring teacher identity issues extensively. The research in a particular field was closely related to the country’s policy and context. So, through Table 6, it can be inferred that the United States, China, and the United Kingdom paid greater attention to teacher identity development and related issues in education. The increasing number of countries paying attention to teacher identity research, such as Australia and the Netherlands, proves that teacher identity research has been a global theme for the past 20 years.

**TABLE 6:** Most relevant countries.

Country	Frequency	Total Citations	Average Article Citations
United States	639	4,906	21.52
United Kingdom	238	2,409	25.09
Netherlands	236	2,122	68.45
Australia	170	1,645	24.19
China	103	1,134	12.33
Canada	89	662	20.69
Portugal	88	626	48.15
Finland	77	619	21.34
Israel	61	293	12.74
Spain	59	251	7.38
Estonia	43	246	35.14
Belgium	40	198	66.00
South Africa	36	179	7.16
Iran	35	162	20.25
New Zealand	34	141	9.40
Cyprus	27	129	16.12
Sweden	27	110	6.88
Ireland	27	107	9.73
Turkey	25	75	5.36
Norway	19	73	8.11

#### Most Relevant Documents

Table 7 presents the top 20 total cited documents and their global citation, normalized total, and normalized global citation. According to Table 7, the *Reconsidering research on teachers’ professional identity* was most wildly cited by having 251 local citations and 1,062 global citations, and it also had 14.6 normalized local citations and 11.79 normalized global citations, which ranked first among all other articles. In this study, Beijaard et al. (2004) reviewed and classified the research of the last decades into three types: (1) studies focusing on the formation of teachers’ professional identity, (2) studies focusing on the characteristic of teachers’ professional identity, and (3) studies focusing on the teachers’ stories for teachers’ professional identity. The four essential features of teachers’ professional identity were listed. They are: (1) professional identity is an ongoing process of interpretation, and reinterpretation of experience (Kerby, 1991), (2) person and context are implied in the professional identity, (3) subidentities are embraced in the teachers’ professional identity, and (4) agency is an essential element in the teachers’ professional identity.

**TABLE 7:** Most relevant documents.

Document	Year	Local citations	Global citations	LC/GC ratio (%)	Normalized local citations	Normalized global citations
Beijaard et al., 2004, Teach Teach Educ	2004	251	1,062	23.63	14.60	11.79
Flores and Day, 2006, Teach Teach Educ	2006	103	546	18.86	6.32	3.05
Akkerman and Meijer, 2011, Teach Teach Educ	2011	92	310	29.68	13.34	7.74
Lasky, 2005, Teach Teach Educ	2005	69	490	14.08	5.75	4.67
Hong, 2010, Teach Teach Educ	2010	45	273	16.48	9.73	8.02
Luehmann, 2007, Sci Educ	2007	37	187	19.79	11.33	3.76
Sutherland et al., 2010, Teach Teach Educ	2010	34	138	24.64	7.36	4.05
Kanno and Stuart, 2011, Mod Lang J	2011	31	113	27.43	4.50	2.82
Lamote and Engels, 2010, Eur J Teach Educ	2010	30	136	22.06	6.49	4.00
Sachs, 2005, J Educ Policy	2001	29	342	8.48	4.68	3.51
Timoštšuk and Ugaste, 2010, Teach Teach Educ	2010	28	108	25.93	6.06	3.17
Thomas and Beauchamp, 2011, Teach Teach Educ	2011	26	150	17.33	3.77	3.75
Day et al., 2005, Teach Teach Educ	2005	25	206	12.14	2.08	1.96
Buchanan, 2015, Teach Teach	2015	23	118	19.49	10.06	7.69
O’Connor, 2008, Teach Teach Educ	2008	22	262	8.40	5.13	5.93
Mockler, 2011, Teach Teach	2011	22	102	21.57	3.19	2.55
Pillen et al., 2013, Eur J Teach Educ	2013	22	98	22.45	5.82	3.68
Urzúa and Vásquez, 2008, Teach Teach Educ	2008	20	94	21.28	4.66	2.13
Clarke, 2009, Educ Philos Theory	2009	20	79	25.32	6.97	2.29
Cohen, 2010, Teach Teach Educ	2010	20	58	34.48	4.33	1.70

However, in this overview, the concept of professional identity was hard to agree with or not defined at all. However, Beijaard et al. (2004) concluded that the studies mainly focused on the teacher’s practical knowledge, but its relationship and professional identity remained unclear. They also directed future research to the study that focuses on the concept of “identity,” “self,” and the “role of context” from more perspectives. The top two most globally cited documents were c*ontext, which shapes and reshapes new teachers’ identities: a multi-perspective study* written by Flores and Day (2006). In this study, they explored how the professional identities of 14 new teachers were shaped and reshaped in their first 2 years of teaching through the interplay between personal, professional, and contextual factors.

Through semi-structured interviews, questionnaires, and teachers’ annual reports, this study found that the three main factors influence the construction, deconstruction, and reconstruction of teachers’ professional identities: (1) prior influences, which means teachers’ previous experiences as pupils; (2) initial teacher training and teaching practice, such as teachers’ motivation and general assessment of their professional learning experiences and its influence on the formation of teachers’ identity; and (3) contexts of teaching, which include the classroom practice, school culture, and leadership. This study concluded that teachers’ personal histories and contextual factors of the workplace have a powerful influence on the construction of teachers’ professional identities and suggested that the influence of pre-service programs might be strengthened by providing more opportunities for teachers’ biographical reflection. Lasky (2005) produced one of the top three most globally cited documents. In *A sociocultural approach to understanding teacher identity, agency, and professional vulnerability in a context of secondary school reform* (2005), Lasky adopted a sociocultural approach to understanding the dynamic interplay among teacher identity, agency, and context. According to this study, the early influences on teacher identity and the current reform context are the primary mediational systems that shape teacher agency and professional vulnerability. Data from interviews and surveys revealed that the political and social context and early teacher development play an essential role in forming a teacher’s professional identity, and there was a gap between teachers’ expectations and the reality of reform.

Moreover, the research suggested that a longitudinal study of how teachers’ identity and agency are mediated over time is essential to explore. Generally, these top local and global cited documents revealed which document had the most significant influence on the perception of a teacher’s professional identity and the production of other works in teacher identity research. Moreover, this table again proves that the journal *Teaching and Teacher Education* was the most relevant institution contributing to teacher identity research because most of these top most influential documents were published on *Teaching and Teacher Education*.

#### Co-citation Analysis of Authors and Institutions

Co-citation networks can illustrate and analyze the performance of authors and institutions in teacher identity research in the past 20 years.

**FIGURE 3:**
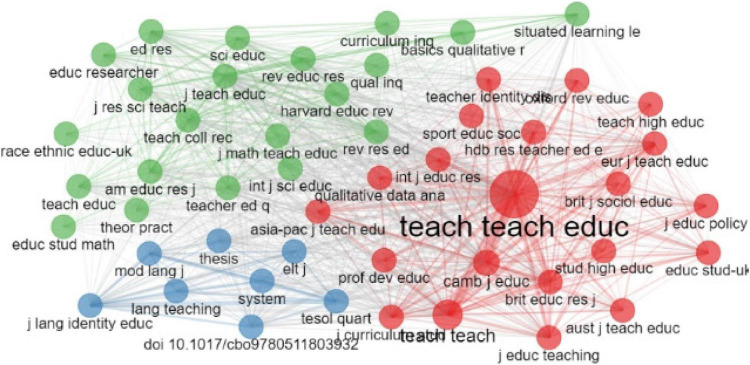
Co-citation of authors.

Figure 3 sheds light on the connection network of authors. A node represents an author, and the node’s size signifies the total number of citations of the authors. A link between the two nodes represents a co-citation relationship, and the thicker the link was, the more frequently the author has been cited. According to the size of nodes and connection between them, this study can conclude that Douwe Beijaard had the most intensive network, followed by Catherine Beauchamp, Christopher Day, Maria Flores, and Sue Lasky, which is in line with the fact that these authors were also the most globally cited author and their documents had a significant influence on teacher identity research. The other two clusters show that the authors in the periphery of the network have also started to publish articles about teacher identity and its related topics, and they have already developed a solid co-citation network. The intensive co-citation networks suggest that the development of teacher identity research involves joint efforts and cooperation among many scholars and researchers.

**FIGURE 4:**
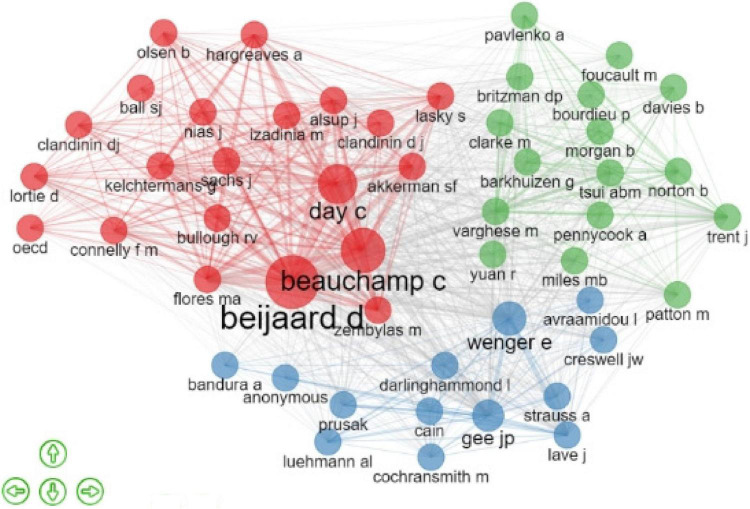
Co-citation of sources.

Figure 4 shows a structure of the co-citation of sources. Similarly, the nodes represent the cited sources, and the node’s size and color signify the source’s citation and connection. To be specific, the larger the node is, the more frequently the source is cited. According to Figure 4, it is well presented that three co-citation clusters were shaped, and each collection had numerous nodes, which shows that these journals were high-cited and had an extensive co-citation network. Among these journals, the journal *Teaching and Teacher Education* had the most intensive network, which shows this journal was most cited and contributed most to the development and progress of teacher identity research. Around the journal *Teaching and Teacher Education*, intensive co-citation networks among many journals, including *Teachers and Teaching European Journal of Teacher Education*, and *Cambridge Journal of Education* are formed. It shows that they were research fronts of teacher identity research and contributed significantly to the progress of teacher identity research. Other sources in the other two clusters indicate that increasingly more authorities paid attention to issues in teacher identity and co-citation network has been formed among them, such as the co-citation network among *Language Teaching, System* and *TESOL Quarterly* in the green cluster, the co-citation network among *Teacher Education Quarterly, Teaching Education*, and *Curriculum Inquiry* in the blue collection. It is proved that the development of teacher identity research was owned by joint efforts of authors and institutions who contributed to the productivity of teacher identity research.

### Research Topics in Teacher Identity Research in the Past 20 Years

For the second question: What were the research topics on teacher identity in the past 20 years? This study intended to answer it through content analysis. Word Cloud, word growth, thematic map, conceptual structure map, and co-occurrence of author’s keywords were used to present research topics in teacher identity research in the past 20 years.

#### Word Cloud

**FIGURE 5:**
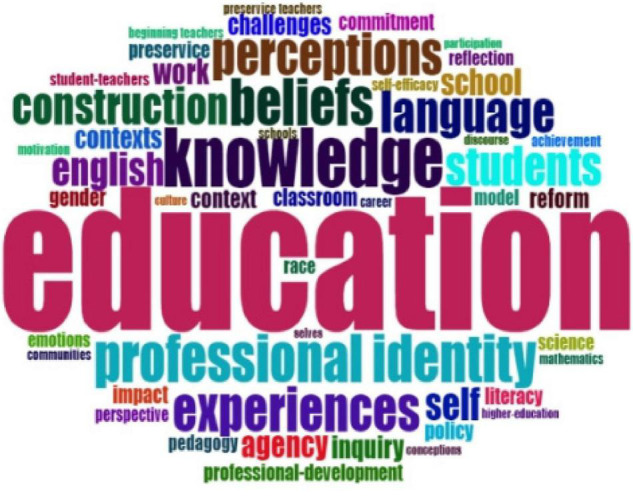
Word Cloud by author’s keywords’ plus.

**FIGURE 6:**
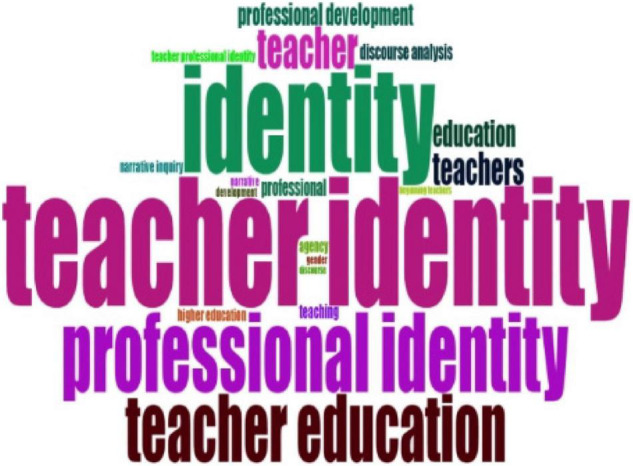
Word Cloud by author’s keywords.

Figures 5, 6 show Word Cloud measured by keywords plus and author’s keywords plus on teacher identity research from 2001 to 2021. With a visual representation of the biblioshiny, words with higher volume and keyword density were displayed in a larger and more prominent font. Word Cloud was applied to analyze the frequently used term to show research topics in teacher identity research. Different words had distinct colors, and the size and position of words represented the frequency of the words. To be specific, the more central the word was, the more frequently it was used. We chose the top 20 words according to keyword plus and author’s keyword plus. First, from Figure 5 of keyword plus mapping, we know that “education” was most frequently used to study teacher identity. It was followed by “knowledge,” “beliefs,” “professional identity,” and “experiences.” This figure shows that teacher identity was an essential research topic in the education field in the past 20 years.

**FIGURE 7:**
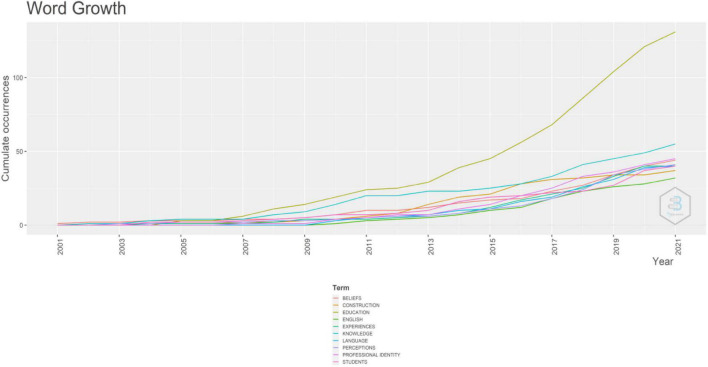
Word growth.

According to Figure 6, shaped by the author’s keyword, “teacher identity” was the most frequently employed keyword in their articles. The items, such as “professional identity,” “identity,” “teacher education,” and the “teacher”, were also intensively used as keywords by authors who studied teacher identity. It shows that they were fundamental topics. Moreover, according to Figure 7, the item “education” employed in the teacher identity increased more quickly, which signifies that teacher identity was an indispensable topic in education research and significantly impacted education development.

#### Thematic Map

**FIGURE 8:**
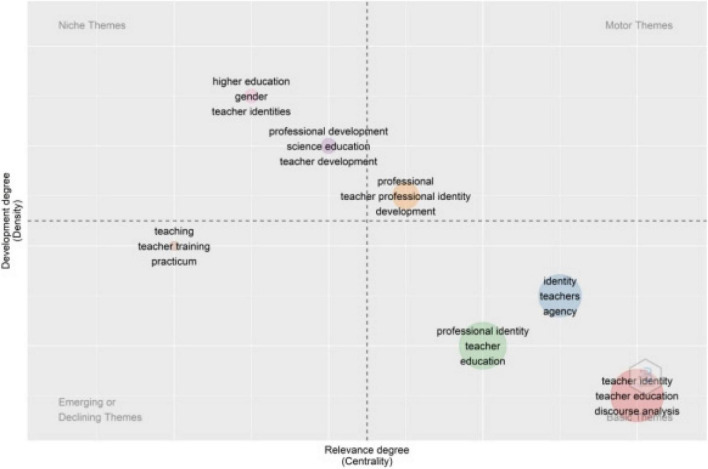
Thematic map.

Figure 8 presents the *status quo* of topic groups in teacher identity research. Different colors represent different clusters, and the keywords in the same collection signify high relatedness. A thematic map is divided into four quadrants according to centrality degree and density. The high centrality and high density in the upper right quadrant indicate well-developed motor themes in the research field. As it can be seen, “professional,” “teacher professional identity,” and “development” were the focus and core of the research and had excellent prospects of development.

The high density and low centrality in the second quadrant indicate niche themes whose development situation is good but has little influence on the research field. It can be seen that “high education,” “gender,” and “curriculum,” and “professional development,” “teacher professional identity,” and “science education” have formed independent research groups. However, their development prospects in the future were uncertain. In the lower-left quadrant, subject clusters had a low centrality and low density. It indicates that various kinds of “teaching training” “teaching” were marginalized. It means that they were emerging or declining topics. The high centrality and low density in the fourth quadrant signified that “teachers,” “identity,” “discourse analysis,” and “teacher education” were the fundamental themes in education. It can be inferred that their theoretical systems were more complete and mature, and these basic themes may provide the theoretical foundation, rationale, and method to teacher identity study. However, their development prospect is not promising.

#### Conceptual Structure Map

**FIGURE 9:**
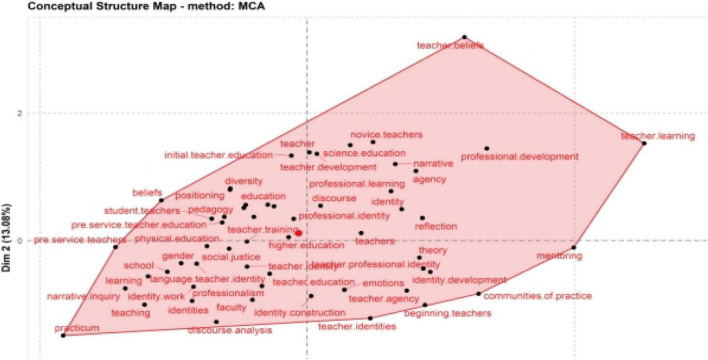
Conceptual structure map.

The biblioshiny for Bibliometrix allows, using the Conceptual Structure Map, to perform multiple correspondence analysis (MCA) to draw a conceptual structure of the field and identify clusters of documents that express common concepts (Aria and Cuccurullo, 2017). MCA makes it possible to conduct a mathematical and graphical investigation of ostensible multivariate information (Greenacre and Blasius, 2006). As seen in Figure 9, the keywords were divided into two clusters using MCA. The colors represent different clusters, the distance between keywords implies the relatedness, the vertex illustrates the word, and the node’s size is proportional to its occurrence. In the red collection, “identity,” “teacher identity,” and “teacher development” frequently appeared. The items “behavior” and “education” were integrated into the blue cluster. Figure 9 shows that a central research theme of teacher identity was formed, and some research topics around teacher identity were relatively well developed. The teachers at various stages, including “initial teacher education,” “novice teacher,” “students teacher,” “pre-service teacher,” and “beginning teacher,” have been studied. The teacher identity of diverse subjects has been intensively explored, including “language teacher identity,” “science teacher,” and “physics teacher.” Furthermore, the teacher identity study was connected to education, including “pedagogy,” “curriculum,” and “theory.” The intersectional and sociocultural features of teacher identity, including agency, social justice, gender in the formation of teacher identity, was an increasingly popular topic.

#### Co-occurrence of Author’s Keywords

**FIGURE 10:**
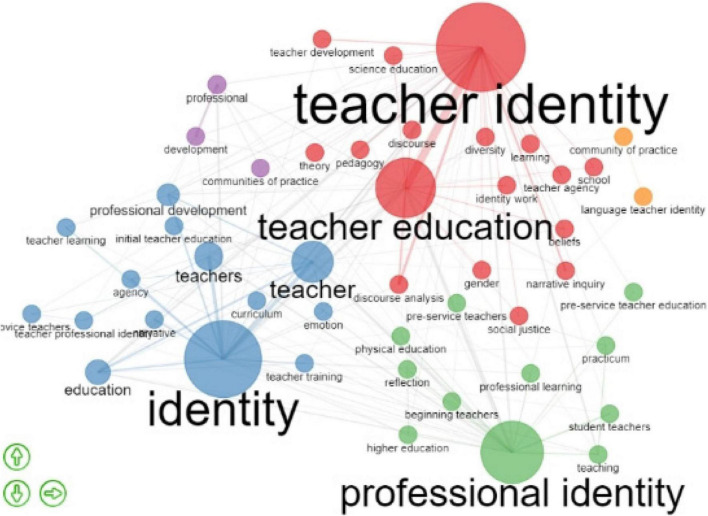
Co-occurrence of author’s keywords.

To understand the research topics of teacher identity in the past 20 years, we have identified that the co-occurrence of keywords analysis is an effective tool to provide an overall knowledge structure. Figure 10 shows the keyword co-occurrence network of publications. The co-occurrence of the author’s keywords suggests the frequency with which keywords, in other words, co-occur in publications. A node represents a keyword, and the bigger the node is, the more citations the keyword has. A link between two nodes represents the co-occurrence of two keywords, and the thicker the line is, the more frequent they co-occur.

Similarly, there were five clusters indicated by five colors in Figure 10. Specifically, “teacher identity” and “teacher education” were prominent in the red cluster and frequently co-occurred. It means that these two topics were emphasized in teacher identity research, and teacher identity research was almost inseparable from teacher education research, the same with “identity” and “teacher” highlighted in the blue cluster. The “personal identity” was prominent in the purple circle, but it did not connect with other words. However, it had a mild relationship with “pre-service teachers,” “students teachers,” and “teaching.” Besides, from the occurrence of the keywords and link between orange and green nodes, this study finds that “language teacher identity” and “community of practice” were usually explored together, and “development” and “professional” research were connected. However, the size of nodes and the thin line between them indicated that they were not researched deeply by now.

In sum, many keywords were employed in the study of teacher identity. “Teacher identity,” “professional identity,” “teacher education,” and “identity” were central and had the highest co-occurrence with other words. It proves that they were fundamental and core research topics in teacher identity research. In the periphery of the network, there was also co-occurrence of other keywords, like “pedagogy,” “teacher agency,” and “teacher training.” It shows that a broader range of research topics in teacher identity research was explored. For all the differences, we can conclude that “teacher identity,” “identity,” and “professional identity” were primary and core research themes, the “beliefs,” “knowledge,” and “experiences” which influence the construction of teacher identity were also popular research contents in the field of teacher identity research.

### Future Research Direction in Teacher Identity Research

For the third question: What are the future research directions in teacher identity? With the help of the bibliometric tool, this article used Thematic Evolution and Thematic Trends to predict future research directions.

#### Thematic Evolution

**FIGURE 11:**
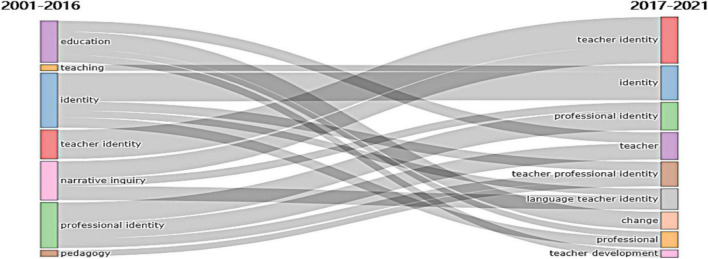
Thematic Evolution by author’s keywords.

Thematic Evolution and Trend Topics can show hot research topics and future research directions. In Thematic Evolution, different themes are represented by different colors, and the rectangle area indicates the degree of research. Figure 11 shows that many research themes are involved in teacher identity research, and the research focus was not static but changed dynamically over time. As time went by, “identity,” “teacher identity,” and “professional identity” remained to be popular research topics. However, the study focus changed from “identity” to “teacher identity,” and the emerging issues, including “teacher,” “professional learning,” “language teacher identity,” and “change” of teacher identity, “teacher professional identity” became more prevalent in 2017–2021. It can be inferred that these topics have a great potential to continue to develop in the future.

#### Trend Topic

**FIGURE 12:**
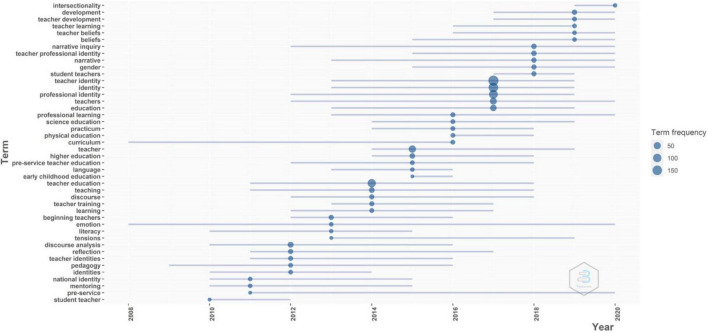
Trend Topics by author’s keywords.

In the Trend Topic, the size of nodes indicates the publication number of topics and the time when the topics were popular. According to the size of blue node in Figure 12, we can get that the research of “teacher identity,” “identity,” and “professional identity” thrived in 2017, indicated by their high productivity. It is again proved that they were fundamental and core topics in teacher identity research in recent years, and they are possible to be paid great attention to in the future. It coincided with the Thematic Evolution in 2017–2021. Besides, recently, emerging topics such as “development,” “teacher development,” and “beliefs” remained popular. The “intersectionality” of teacher identity was an emerging research topic that occurred in 2019, and it would keep as a promising research topic in the future, indicated by its high productivity in 2020. It can be concluded that these topics, including “teacher identity,” “identity,” “professional identity,” “development,” “teacher development,” “beliefs,” and “intersectionality,” have a great potential to thrive in teacher identity research in the future.

## Conclusion and Implication

This study employed a bibliometric tool to analyze the 20-year development of teacher identity research in a detailed manner. Using the visualization method’s productivity analysis, content analysis, and citation analysis, we acquired information about research status and research topics of teacher identity research in the past 20 years. Moreover, future research directions were predicted to enlighten scholars and researchers interested in the issues related to teacher identity research.

First, through the bibliometric analysis in 20 years of development of teacher identity research, this study concluded that teacher identity is an encompassing concept and remains a popular topic in the academic field. Among the numerous research topics, “teacher identity,” “professional identity,” and “identity” were the fundamental and core themes. “Education,” “knowledge,” “beliefs,” “agency,” and “experiences”, which shape that teacher identity was also active and popular topic in the teacher identity research. Teacher identity was an everlasting research theme that involved diverse research topics in the past 20 years.

Second, teacher identity studies involved many authors, institutions, sources, and countries, and the cooperation mainly conducted its survey among different authors and institutions in other countries. Douwe Beijaard, Maria Flores, and Sun Lasky played a crucial role in teacher identity research as their documents contributed a lot to the production of other research articles. An increasing number of researchers devoted themselves to teacher identity research indicates that teacher identity research in education has practical significance for many aspects of human society. In terms of the documents, the documents *Reconsidering research on teacher’s professional identity* (Beijaard et al., 2004), *Context, which shapes and reshapes new teachers’ identities: a multi-perspective study* (Flores and Day, 2006), and *A sociocultural approach to understanding teacher identity, agency, and professional vulnerability in a context of secondary school reform* (Lasky, 2005) were the three most essential documents in teacher identity research. The journal *Teachers and Teacher Education*, *Teachers and Teaching*, and *European Journal of Teachers Education* were the institutions that had the most significant impact on the study of teacher identity. The United States, United Kingdom, and China were the top three countries that contributed to the development of teacher identity research, and strong collaborations and networks among them were formed. These aspects in the teacher identity research are closely connected as the most cited person contributes to the formation of the countries, institutions, and articles that have considerable influences on teacher identity research. All types of this information benefit greatly to stakeholders in the education field. It may help novice and experienced researchers find the most relevant knowledge about teacher identity research and look for potential cooperators in this field. Moreover, it can provide policymakers with a standard to evaluate the performance of institutions, universities, and authors in teacher identity research.

Third, through an analysis of Thematic Evolution and Trend Topic, we can conclude that fundamental and core topics in teacher identity research, including “identity,” “teacher identity,” and “professional identity,” are possible to be paid more attention to in the future, which coincides with the Thematic Evolution in 2017–2021. Besides, recently, the emerging topics, including “development,” “teacher development,” and “beliefs” remained popular topics. Remarkably, the “intersectionality” of teacher identity, which shows the influence of gender, social justice, race, and other sociocultural factors on the construction and reconstruction of teacher identity, is an emerging and promising topic in the future indicated by its high productivity. It can be concluded that these topics have the potential to thrive in teacher identity research in the future. This trend agrees with the postmodern stance on teacher identity because it holds that the construction and reconstruction of teacher identity result from the interaction between the individual and context. Moreover, teacher identity development is a relational phenomenon, and sociocultural factors offer a more practicable perspective on teacher identity development.

Although bibliometric methods effectively reveal a research field’s knowledge structure and research topics and can predict future research directions, it is not a substitute for extensive reading. The broad task remains a premise for an in-depth understanding of the research field. Furthermore, in the future, the quantitative method in the teacher identity should be applied more to challenge the psychological feature of methodology in the research. Moreover, an integrated method uses qualitative and quantitative methods in future teacher identity research.

## Limitation

As a quantitative study with a bibliometric tool based on the data collected from a database, the focus is on the presentation of pictures and statistics, but the qualitative method for data collection and data analysis is absent. Furthermore, it is a review study providing an overall view of teacher identity research in the past 20 years based on the bibliometric analysis, but it has not delved into a specific aspect of teacher identity to give a thorough investigation. To provide a more extensive understanding of teacher identity, we insist that future research could expand the study to employ a variety of data collection to explore more specific issues in teacher identity research.

## Data Availability Statement

The original contributions presented in the study are included in the article/supplementary material, further inquiries can be directed to the corresponding author.

## Author Contributions

YZ and PW have shared the equal contributions. PW found the research topic and modified the manuscript several times. YZ collected, analyzed the data, and completed the manuscript under the supervision of PW. Both authors have contributed to the article and approved the submitted version.

## Conflict of Interest

The authors declare that the research was conducted in the absence of any commercial or financial relationships that could be construed as a potential conflict of interest.

## Publisher’s Note

All claims expressed in this article are solely those of the authors and do not necessarily represent those of their affiliated organizations, or those of the publisher, the editors and the reviewers. Any product that may be evaluated in this article, or claim that may be made by its manufacturer, is not guaranteed or endorsed by the publisher.

## References

[B100] Akkerman S. F. Meijer P. C. (2011). A dialogical approach to conceptualizing teacher identity. *Teach. Teacher Educ.* 27, 308–319. 10.1016/j.tate.2010.08.013

[B1] Aria M. Cuccurullo C. (2017). bibliometrix : an R-tool for comprehensive science mapping analysis. *J. Informetr.* 11 959–975. 10.1016/j.joi.2017.08.007

[B2] Baker H. K. Kumar S. Pandey N. (2020). A bibliometric analysis of managerial finance: a retrospective. *Manage. Finance* 46 1495–1517. 10.1108/mf-06-2019-0277

[B3] Beauchamp C. Thomas L. (2009). Understanding teacher identity: an overview of issues in the literature and implications for teacher education. *Cambr. J. Educat.* 39 175–189. 10.1080/03057640902902252

[B4] Beijaard D. Meijer P. C. (2017). “Developing the personal and professional in making a teacher identity,” in *The SAGE Handbook of Research on Teacher Education*, eds Clandinin J. D. Jukka H. (London: Sage), 177–192. 10.4135/9781526402042.n10

[B5] Beijaard D. Meijer P. C. Verloop N. (2004). Reconsidering research on teachers’ professional identity. *Teach. Teach. Educat.* 20 107–128. 10.1016/j.tate.2003.07.001

[B6] Birkle C. Pendlebury D. A. Schnell J. Adams J. (2020). Web of Science as a data source for research on scientific and scholarly activity. *Quant. Sci. Stud.* 1 363–376. 10.1162/qss_a_00018

[B7] Bonilla C. A. Merigó J. M. Torres-Abad C. (2015). Economics in Latin America: a bibliometric analysis. *Scientometrics* 105 1239–1252. 10.1007/s11192-015-1747-7

[B8] Buchanan R. (2015). Teacher identity and agency in an era of accountability. *Teach. Teach.* 21 700–719. 10.1080/13540602.2015.1044329

[B9] Chiu W.-T. Ho Y.-S. (2007). Bibliometric analysis of tsunami research. *Scientometrics* 73 3–17. 10.1007/s11192-005-1523-1 PMC7089365 32214553

[B10] Clarke M. (2009). The ethico-politics of teacher identity. *Educ. Philos. Theory* 41 185–200. 10.1111/j.1469-5812.2008.00420.x

[B11] Cohen J. L. (2010). Getting recognised: teachers negotiating professional identities as learners through talk. *Teach. Teach. Educ.* 26 473–481. 10.1016/j.tate.2009.06.005

[B12] Day C. Elliot B. Kington A. (2005). Reform, standards and teacher identity: challenges of sustaining commitment. *Teach. Teach. Educ.* 21 563–577. 10.1016/j.tate.2005.03.001

[B13] Derviş H. (2019). Bibliometric analysis using Bibliometrix an R package. *J. Scientometric Res.* 8 156–160. 10.5530/jscires.8.3.32

[B14] Diodato V. (1994). *Dictionary of Bibliometrics.* New York, NY: Haworth Press.

[B15] Ellegaard O. Wallin J. A. (2015). The bibliometric analysis of scholarly production: How great is the impact? *Scientometrics* 105 1809–1831. 10.1007/s11192-015-1645-z 26594073 PMC4643120

[B16] Flores M. A. Day C. (2006). Contexts which shape and reshape new teachers’ identities: a multi-perspective study. *Teach. Teacher Educat.* 22 219–232. 10.1016/j.tate.2005.09.002

[B17] Frandsen T. F. Rousseau R. (2005). Article impact calculated over arbitrary periods. *J. Am. Soc. Inform. Sci. Technol.* 56 58–62. 10.1002/asi.20100

[B18] Gautam P. Maheshwari S. Kaushal-Deep S. M. Bhat A. R. Jaggi C. K. (2020). COVID-19: a Bibliometric Analysis and Insights. *Int. J. Math. Engin. Manag. Sci.* 5 1155–1169. 10.33889/ijmems.2020.5.6.088

[B19] Greenacre M. Blasius J. (2006). *Multiple Correspondence Analysis and related Methods.* Milton Park: Taylor and Francis Group, 10.1201/9781420011319

[B20] Hao L. (2018). 国际科研评价研究的科学计量及可视化分析——基于 Biblioshiny 程序. *图书情报导刊* 12 36–44.

[B21] Hong J. Y. (2010). Pre-service and beginning teachers’ professional identity and its relation to dropping out of the profession. *Teach. Teach. Educ.* 26 1530–1543. 10.1016/j.tate.2010.06.003

[B22] Izadinia M. (2014). Teacher educators’ identity: a review of literature. *Eur. J Teach. Educat.* 37 426–441. 10.1080/02619768.2014.947025

[B23] Jupp J. C. Lensmire T. J. (2016). Second-wave white teacher identity studies: toward complexity and reflexivity in the racial conscientization of white teachers. *Int. J. Qual. Stud. Educ.* 29 985–988. 10.1080/09518398.2016.1189621

[B24] Kanno Y. Stuart C. (2011). Learning to become a second language teacher: identities-in-practice. *Mod. Lang. J.* 95 236–252. 10.1111/j.1540-4781.2011.01178.x

[B25] Koseoglu M. A. Rahimi R. Okumus F. Liu J. (2016). Bibliometric studies in tourism. *Ann. Tour. Res.* 61 180–198. 10.1016/j.annals.2016.10.006

[B26] Lamote C. Engels N. (2010). The development of student teachers’ professional identity. *Eur. J. Teach. Educ.* 33 3–18. 10.1080/02619760903457735

[B27] Lasky S. (2005). A sociocultural approach to understanding teacher identity, agency and professional vulnerability in a context of secondary school reform. *Teach. Teach. Educ.* 21 899–916. 10.1016/j.tate.2005.06.003

[B28] Li K. Rollins J. Yan E. (2018). Web of Science use in published research and review papers 1997–2017: a selective, dynamic, cross-domain, content-based analysis. *Scientometrics* 115 1–20. 10.1007/s11192-017-2622-5 29527070 PMC5838136

[B29] Lopes A. Pereira F. (2012). Everyday life and everyday learning: the ways in which pre-service teacher education curriculum can encourage personal dimensions of teacher identity. *Eur. J. Teach. Educat.* 35 17–38. 10.1080/02619768.2011.633995

[B30] Luehmann A. L. (2007). Identity development as a lens to science teacher preparation. *Sci. Educ.* 91 822–839. 10.1002/sce.20209

[B31] Mockler N. (2011). Beyond ‘what works’: understanding teacher identity as a practical and political tool. *Teach. Teach.* 17 517–528. 10.1080/13540602.2011.602059

[B32] Moral-Muñoz J. A. Herrera Viedma E. Santisteban Espejo A. Cobo M. J. (2020). Software tools for conducting bibliometric analysis in science: an up-to-date review. *Dialnet* 29:e290103. 10.1200/CCI.19.00042 31545655 PMC6873946

[B33] Nafade V. Nash M. Huddart S. Pande T. Gebreselassie N. Lienhardt C. (2018). A bibliometric analysis of tuberculosis research, 2007–2016. *PLoS One* 13:e0199706. 10.1371/journal.pone.0199706 29940004 PMC6016906

[B34] O’Connor K. E. (2008). “You choose to care”: teachers, emotions and professional identity. *Teach. Teach. Educ.* 24 117–126. 10.1016/j.tate.2006.11.008

[B35] Pennington M. C. (2014). “Teacher identity in TESOL: a frames perspective,” in *Advances and Current Trends in Language Teacher Identity Research*, eds Cheung Y. L. Said S. B. Park K. (Milton Park: Routledge), 16–30.

[B36] Pillen M. Beijaard D. Brok P. D. (2013). Tensions in beginning teachers’ professional identity development, accompanying feelings and coping strategies. *Eur. J. Teach. Educ.* 36 240–260. 10.1080/02619768.2012.696192

[B37] Pritchard A. (1969). Statistical bibliography or bibliometrics. *J. Doc.* 25:348.

[B38] Sachs J. (2005). “Teacher education and the development of professional identity: learning to be a teacher,” in *Connecting Policy and Practice: Challenges for Teaching and Learning in Schools and Universities*, eds Kompf M. Denicolo P. (New York, NY: Routledge), 5–21.

[B39] Schutz P. A. Francis D. C. Hong J. (eds) (2018). “Research on teacher identity: introduction to mapping challenges and innovations,” in *Research on teacher identity*, (Cham: Springer), 3–9. 10.1007/978-3-319-93836-3_1

[B40] Sutherland L. Howard S. Markauskaite L. (2010). Professional identity creation: examining the development of beginning preservice teachers’ understanding of their work as teachers. *Teach. Teach. Educ.* 26 455–465. 10.1016/j.tate.2009.06.006

[B41] Thomas L. Beauchamp C. (2011). Understanding new teachers’ professional identities through metaphor. *Teach. Teach. Educ.* 27 762–769. 10.1016/j.tate.2010.12.007

[B42] Timoštšuk I. Ugaste A. (2010). Student teachers’ professional identity. *Teach. Teach. Educ.* 26 1563–1570. 10.1016/j.tate.2010.06.008

[B43] Trent J. (2010). Teacher education as identity construction: insights from action research. *J. Educat. Teach.* 36 153–168. 10.1080/02607471003651672

[B44] Trent J. (2012). Becoming a teacher: the identity construction experiences of beginning English language teachers in Hong Kong. *Austr. Educat. Res.* 39 363–383. 10.1007/s13384-012-0067-7

[B45] Trent J. (2013). From learner to teacher: practice, language, and identity in a teaching practicum. *Asia Pacific J. Teacher Educat.* 41 426–440. 10.1080/1359866x.2013.838621

[B47] Urzúa A. Vásquez C. (2008). Reflection and professional identity in teachers’ future-oriented discourse. *Teach. Teach. Educ.* 24 1935–1946. 10.1016/j.tate.2008.04.008

[B48] van Lankveld T. Schoonenboom J. Volman M. Croiset G. Beishuizen J. (2016). Developing a teacher identity in the university context: a systematic review of the literature. *Higher Educat. Res. Devel.* 36 325–342. 10.1080/07294360.2016.1208154

[B49] Yuan R. Burns A. (2016). Teacher identity development through action research: a Chinese experience. *Teachers Teach.* 23 729–749. 10.1080/13540602.2016.1219713

[B50] Yuan R. Lee I. (2014). The cognitive, social and emotional processes of teacher identity construction in a pre-service teacher education programme. *Res. Papers Educat.* 30 469–491. 10.1080/02671522.2014.932830

[B51] Yuan R. Lee I. (2016). I need to be strong and competent’: a narrative inquiry of a student-teacher’s emotions and identities in teaching practicum. *Teachers Teach.* 22 819–841. 10.1080/13540602.2016.1185819

[B52] Yuan R. Mak P. (2018). Reflective learning and identity construction in practice, discourse and activity: Experiences of pre-service language teachers in Hong Kong. *Teaching Teacher Educat.* 74 205–214.

[B53] Zupic I. Èater T. (2015). Bibliometric methods in management and organization. *Organ. Res. Methods* 18 429–472. 10.1177/1094428114562629

